# Black-legged kittiwakes as messengers of Atlantification in the Arctic

**DOI:** 10.1038/s41598-017-19118-8

**Published:** 2018-01-19

**Authors:** Mikko Vihtakari, Jorg Welcker, Børge Moe, Olivier Chastel, Sabrina Tartu, Haakon Hop, Claus Bech, Sébastien Descamps, Geir Wing Gabrielsen

**Affiliations:** 1 Norwegian Polar Institute, Fram Centre, NO-9296 Tromsø, Norway; 2 BioConsultSH, Schobüller Str. 36, DE-25813 Husum, Germany; 3 Norwegian Institute for Nature Research, Pb 5685 Sluppen, NO-7485 Trondheim, Norway; 4 Centre d’Etudes Biologiques de Chizé, UMR 7372 – CNRS & Université de La Rochelle, FR-79360 Villiers en Bois, France; 5 Department of Arctic and Marine Biology, Faculty of Biosciences, Fisheries and Economics, UiT The Arctic University of Norway, NO-9037 Tromsø, Norway; 6 Department of Biology, Norwegian University of Science and Technology (NTNU), NO-7491 Trondheim, Norway

## Abstract

Climate warming is rapidly altering marine ecosystems towards a more temperate state on the European side of the Arctic. However, this “Atlantification” has rarely been confirmed, as long-term datasets on Arctic marine organisms are scarce. We present a 19-year time series (1982–2016) of diet samples from black-legged kittiwakes as an indicator of the changes in a high Arctic marine ecosystem (Kongsfjorden, Svalbard). Our results highlight a shift from Arctic prey dominance until 2006 to a more mixed diet with high contribution of Atlantic fishes. Capelin, an Atlantic species, dominated the diet composition in 2007, marking a shift in the food web. The occurrence of polar cod, a key Arctic fish species, positively correlated with sea ice index, whereas Atlantic species demonstrated the opposite correlation indicating that the diet shift was likely connected with recent climate warming. Kittiwakes, which gather available fish and zooplankton near the sea surface to feed their chicks, can act as messengers of ecosystem change. Changes in their diet reveal that the Kongsfjord system has drifted in an Atlantic direction over the last decade.

## Introduction

**Figure 1:**
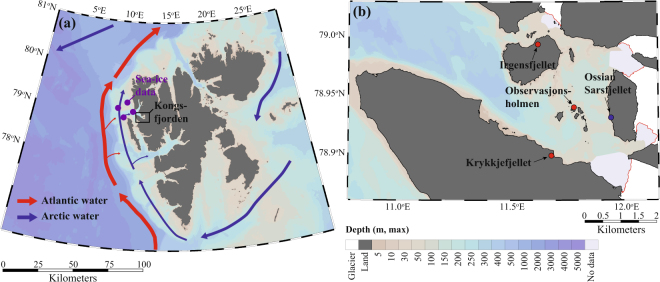
(**a**) Overview map of Svalbard showing Kongsfjorden, where kittiwake regurgitates were collected (black frame), West Spitsbergen Current transporting warm and saline Atlantic water as red arrows, cold Arctic currents as blue arrows, and centers for NASA sea ice concentration data blocks (purple dots). (**b**) Detailed map of Kongsfjorden showing the sampled colonies (red dots), the colony for population size estimates (blue dot), and tidewater glacier fronts in 2009 highlighted with red lines. Colors in the ocean indicate depth and are plotted with same depth scale for both sub-figures. The figure was created using Ocean Data View^72^.

Climate change in the Arctic is not only a model projection but an ongoing process^1,2^. The European side of the Arctic, including the northern Barents Sea and Svalbard archipelago (Fig. 1a), has gradually experienced less sea ice and warmer water temperatures during the last four decades^3–5^. Such changes in physical drivers influence species distributions: Arctic species retract northwards following the retreating marginal ice zone, while abundances of boreal and Atlantic species are expected to increase in the warmer sea-ice-free habitat^6–8^. This “borealization” of the Arctic^6^, or more specifically, the “Atlantification” of the European Arctic, will affect the food web structure by altering the community composition of lower and middle trophic levels^6,7,9^. Appearances of such changes in empirical data depend on temporal and spatial scales of examination as well as the strength of drivers causing the change. On large spatial- or long time-scales, community changes may occur in a fluctuating pattern^10^. In smaller spatial-, shorter time-scales, or in case of sufficiently high driver impact, shifts towards a warmer state might be seen as abrupt changes – or “regime-shifts” – defined as abrupt and persistent changes in the ecosystem structure as a response to changing environmental conditions^11–13^. Documenting ecosystem shifts on different scales is challenging in the Arctic marine environment, because long-term monitoring programs are rare and long-term data on abundances scarce^9^.

Many surface feeding seabirds forage in the pelagic ecosystem and can therefore act as bioindicators of changes in pelagic communities. Diet samples collected from these birds can be used as ecosystem data from locations that are otherwise difficult to access^14–16^. Black-legged kittiwakes (*Rissa tridactyla*, Linnaeus 1758), referred to as “kittiwakes” hereafter, are small colonial gulls with a circumpolar distribution and especially well-suited for such studies. Diet samples can be collected directly from kittiwake breeding colonies as regurgitates^17^. Kittiwakes do not have a highly specialized diet, but feed virtually on all small-sized prey available near the sea-surface – prey items including small fish, crustaceans and other planktonic invertebrates^17,18^. In Kongsfjorden (ca. 79° N 12° E), a glacially-influenced fjord on the west coast of Spitsbergen, kittiwakes exhibit relatively predictable foraging behavior during the breeding period and almost all feeding occurs within the fjord^19–21^. Consequently, the incubation and chick-rearing diet of kittiwakes in Kongsfjorden primarily reflects prey availability within the fjord, and, thus, the composition of diet samples may be used as an indicator of ecosystem changes in this area^15,16^.

Kongsfjorden is an ideal model fjord to study Arctic warming. The fjord is located in the high-Arctic and is influenced by two ocean currents. The West Spitsbergen Current (WSC), a branch of the North Atlantic Current, transports large amounts of relatively warm and saline Atlantic Water and a coastal current on the shelf transports cold and less saline Arctic Water (Fig. 1a)^22^. These two currents mix at the shelf-break creating a dynamic in-fjord hydrography with conditions fluctuating from year to year^4,23^. Consequently, the fjord harbors a mixture of Atlantic and Arctic fauna, the composition of which varies among years depending on advection of Transformed Atlantic Water, which represents a mix of the Atlantic and Arctic Water masses^24,25^. In recent years, Kongsfjorden has been strongly influenced by the WSC with increasing temperatures and declining sea ice cover^4^. Consequently, Arctic species (e.g. polar cod *Boreogadus saida* and the hyperiid amphipod *Themisto libellula*) are expected to be, at least partly, replaced by species of sub-Arctic and Atlantic origin, such as capelin (*Mallotus villosus*), Atlantic herring *(Clupea harengus*, referred to as “herring”), Atlantic cod (*Gadus morhua*) and haddock (*Melanogrammus aeglefinus*)^26,27^. In this study, we used a unique long-term time series of diet samples from kittiwakes breeding around inner Kongsfjorden to test whether the ecosystem has changed in an Atlantic direction, with concurrent food-web changes in the lower part of the marine ecosystem.

## Results

### Changes in diet composition

**Figure 2:**
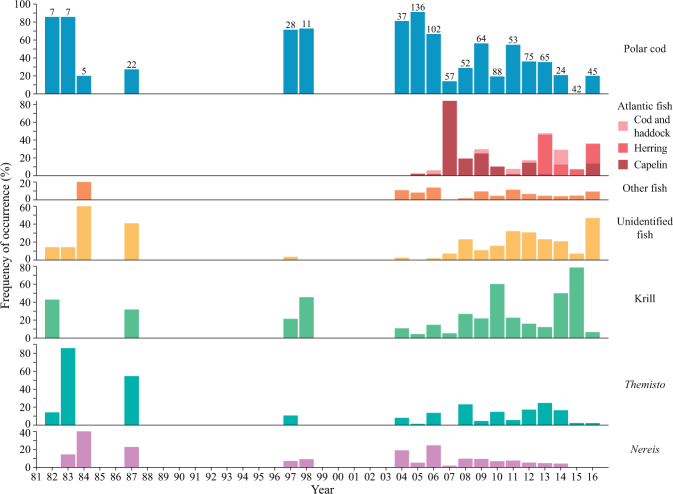
Frequency of occurrence of the major diet items in kittiwake regurgitates sampled from Kongsfjorden 1982−2016. Other fishes comprise mostly glacier lanternfish and white barracudina, whereas krill and *Themisto* consist almost solely of *Thysanoessa inermis* and *Themisto libellula*, respectively (see Extended Data Table 1). Number of diet samples included in the analysis is given on top of each bar for polar cod.

Polar cod, the krill *Thysanoessa inermis*, and capelin were the most frequent prey species in kittiwake regurgitates (Table 1). Fish dominated the diet composition in all years except for 2010 and 2015 when *T*. *inermis* contributed most to the diet (Fig. 2, Extended Data Table 1). In general, polar cod was the most important fish species in the diet (Table 1), but its contribution decreased significantly over the years (linear mixed model, LMM: −5.4% AP/year, t = −14.1, df = 877, p < 0.01). Consequently, Atlantic fish species (capelin, herring, Atlantic cod, and haddock) became more important diet items than polar cod in 2007 and 2013–2016 (Fig. 2, Extended Data Table 1).

**Table 1:** Overview of all encountered diet items in kittiwake regurgitates.

Diet item	FO (%)	N	WW (g)	MM (g)	AP (%)
Fish					
Polar cod (*Boreogadus saida*)	47.7	419	8.2 (0.4)	17.2 (0.7)	42.2 (1.6)
Atlantic fishes					
Capelin (*Mallotus villosus*)	12.2	107	1.9 (0.2)	15.7 (1.2)	10.0 (1.0)
Atlantic herring (*Clupea harengus*)	5.1	45	0.6 (0.1)	12.1 (1.4)	4.0 (0.6)
Atlantic cod (*Gadus morhua*)	1.5	13	0.2 (0.1)	16.3 (4.2)	0.9 (0.3)
Haddock (*Melanogrammus aeglefinus*)	0.5	4	0.0 (0.0)	6.7 (0.6)	0.3 (0.2)
Other fishes					
Glacier lanternfish (*Benthosema glaciale*)	3.5	31	0.4 (0.1)	10.4 (2.8)	2.4 (0.5)
White barracudina (*Arctozenus risso*)	1.8	16	0.2 (0.1)	10.5 (1.9)	1.1 (0.3)
Daubed shanny (*Leptoclinus maculatus*)	1.1	10	0.0 (0.0)	3.6 (0.5)	0.3 (0.1)
Snake blenny (*Lumpenus lampretaeformis*)	0.3	3	0.0 (0.0)	3.0 (0.2)	0.1 (0.1)
Rockfish (*Sebastes* spp.)	0.2	2	0.1 (0.0)	24.6 (5.2)	0.2 (0.1)
Shorthorn sculpin (*Myoxocephalus scorpius*)	0.1	1	0.0 (0.0)	21.5	0.1 (0.1)
Threespot eelpout (*Lycodes rossi*)	0.1	1	0.0 (0.0)	8.1	0.0 (0.0)
Unidentified fish	14.2	125	1.3 (0.1)	8.8 (0.7)	12.0 (1.1)
Crustacea					
Krill					
*Thysanoessa inermis*	22.6	199	1.8 (0.2)	8.0 (0.6)	17.4 (1.2)
*Thysanoessa longicaudata*	0.3	3	0.0 (0.0)	2.4 (1.9)	0.1 (0.1)
Amphipods					
*Themisto libellula*	8.9	78	0.2 (0.1)	2.8 (0.6)	3.0 (0.5)
*Themisto abyssorum*	1.3	11	0.0 (0.0)	2.1 (0.8)	0.5 (0.2)
Shrimp					
Northern prawn (*Pandalus borealis*)	2.3	20	0.0 (0.0)	1.2 (0.3)	0.2 (0.1)
Crimson pasiphaeid (*Pasiphaea tarda*)	1.3	11	0.0 (0.0)	0.5 (0.1)	0.2 (0.1)
Unidentified Crustacea	2.6	23	0.0 (0.0)	0.7 (0.1)	0.5 (0.2)
Other					
Polychaetes (*Nereis* spp.)	8.2	72	0.4 (0.1)	4.9 (0.9)	3.2 (0.5)
Pteropod (*Limacina helicina*)	1	9	0.1 (0.0)	5.5 (1.8)	0.8 (0.3)
Chaetognath (*Parasagitta elegans*)	0.9	8	0.0 (0.0)	2.1 (1.1)	0.4 (0.2)
Cephalopods	0.1	1	0.0 (0.0)	0.8	0.0 (0.0)
Trawler waste	0.5	4	0.0 (0.0)	5.5 (2.5)	0.2 (0.1)

Columns from the left: The common and scientific name as well as group and sub-group of a diet item used in text; frequency of occurrence (FO, %); number of kittiwake regurgitates where the given diet item was present (N); average wet weight (WW, grams); average meal mass (MM, grams); and average mass percentage (AP, %). Data are combined over 1997–2016. Standard error of mean is given in parenthesis. Total number of samples was 879. Colored symbols indicate division of taxa related to their origin ( = Arctic,  = Atlantic,  = mesopelagic and  = intermediate).

**Figure 3:**
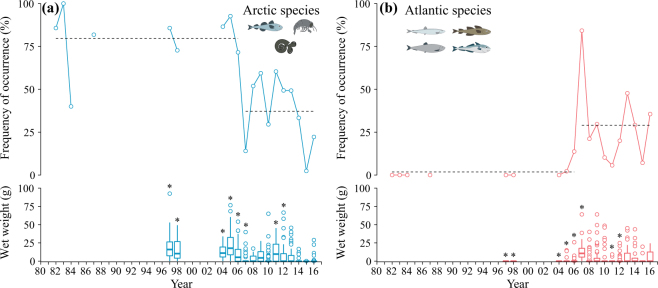
Frequency of occurrence (lines) and wet weight (boxplots) of Arctic (blue, a) and Atlantic (red, b) species throughout the study. Dashed lines indicate the two regimes identified by the change-point analysis (Table 2, Text S1). Stars above boxplots indicate years when wet weight of Arctic and Atlantic taxa differed significantly from each other (Extended Data Table 2). Boxplots display median (thick line), 25% and 75% quantiles (edges of boxes), 1.5 × inter-quantile range (whiskers) as well as outliers. Boxplots are based on wet weights from individual diet samples (i.e. birds). The icons in the figure were partly made by Dr. Malin Daase.

Arctic species consisted mostly of polar cod and the amphipod *Themisto libellula*, whereas capelin and herring were the major Atlantic species in the dataset (Table 1). Change-point analysis indicated a shift in occurrence of Arctic and Atlantic species with Arctic species becoming less frequent from 2007 onwards (Fig. 3, Table 2). Arctic species contributed significantly more than Atlantic species to the wet weight of regurgitates in 1997–1998, 2004–2006, and 2011–2012 (Fig. 3, see Extended Data Table 2 for model statistics), whereas Atlantic species contributed more than Arctic species in 2007. After this year, the contribution of Atlantic species decreased until 2012, and increased again in 2013 leading to a dominance of Atlantic species over Arctic counterparts, although this difference was not statistically significant (Extended Data Table 2).

**Table 2:** Best fitting models for Arctic and Atlantic species frequency of occurrence as well as explanatory variable time-series. Model type is given in “Type” column. “Est.” column gives the parameter estimates for model terms specified in the “Term” column.

Variable	Type	Term	Est.	Df	Stat.	P
Diet data						
Arctic species	Constant change-point			17	96.0	<0.001
	Intercept 1982–2006		78.1		12.4	<0.001
	Intercept 2007–2016		37.2		6.2	<0.001
Atlantic species	Constant change-point			17	14.0	<0.001
	Intercept 1982–2006		1.8		0.3	0.763
	Intercept 2007–2016		29.0		5.3	<0.001
Environmental data						
Sea ice index	Linear			18	19.4	<0.001
	Intercept 1997–2016		19.9		6.2	<0.001
	Slope 1997–2016		−0.5		−4.4	<0.001
Temperature	Linear			18	12.6	0.002
	Intercept 1997–2016		0.3		0.3	0.731
	Slope 1997–2016		0.1		3.5	0.002
Demographic data						
Population size	Linear interaction change-point			15	5.6	0.009
	Intercept 1997–2006		167.8		1.9	0.081
	Slope 1997–2006		−6.9		−2.5	0.022
	Intercept 2007–2016		80.1		1.1	0.304
	Slope 2007–2016		1.4		0.6	0.552
Clutch size	Constant			17		
	Intercept 1997–2015		1.7		44.7	<0.001
Breeding success	Constant			15		
	Intercept 1997–2014		0.8		8.8	<0.001

“Df” column indicates residual degrees of freedom. “Stat.” column report *t* values for model terms and *F* values for entire models. “P” column specifies the respective *p* value. See Text S1 and Table S1 for model selection, and Fig. 3 and Fig. 5 for graphical presentation of the models.

**Figure 4:**
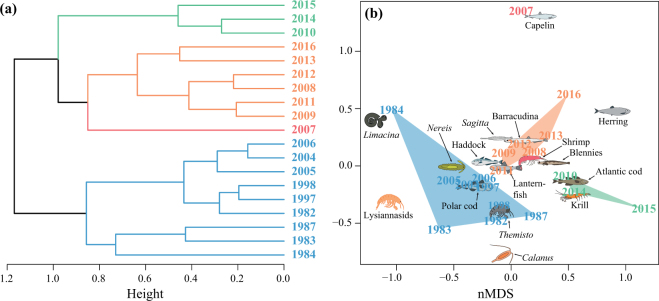
Hierarchical cluster analysis (**a**) and non-metric multidimensional scaling (nMDS, b) of annual frequency of occurrence data. Icons indicate species averages in (**b**). The icons in the figure were partly made by Dr. Malin Daase.

Hierarchical clustering of sampling years using frequency of occurrence revealed similar change in species composition of regurgitates from 2007 (Fig. 4). The years between 1982 and 2006 were grouped together due to a high occurrence of Arctic species such as polar cod (FO: 20–91%), *Themisto libellula*, *Nereis* spp. and *Limacina helicina*. The year 2007 was dominated by capelin (FO: 84%), marking a shift in the diet dataset. The years 2008–2009, 2011–2013 and 2016 were grouped together due to occurrence of Atlantic fish species, such as capelin and herring. Finally, the years 2010 and 2014–2015 were characterized by high occurrence of krill (FO: 54–79%), almost solely *Thysanoessa inermis* (Extended Data Table 1).

### Changes in explanatory variables

**Figure 5:**
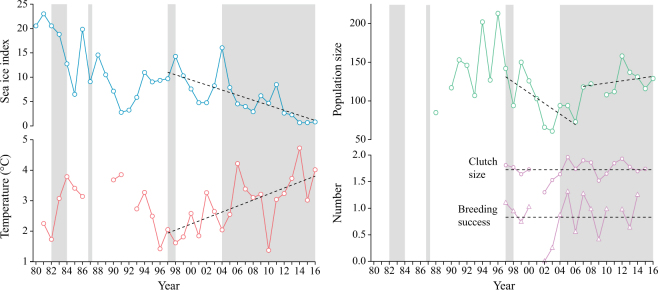
Sea ice index (blue) at Northwest Spitsbergen, temperature (red), breeding population size (green), clutch size and breeding success (purple) in Kongsfjorden from 1980 until 2016. Grey shading illustrates years with kittiwake regurgitates. Dashed lines indicate the best fitting linear models as indicated by the change-point analysis and model selection (Table 2, Text S1).

Sea ice index decreased linearly from 1997 until 2016, while temperature in Kongsfjorden showed the opposite trend (Fig. 5, Table 2). Kittiwake population size decreased from 1997 until 2006, demonstrated a step increase in 2007, and remained stable thereafter (Table 2). Clutch size or breeding success did not show any significant linear trends over time (Fig. 5).

### Environmental correlations

**Figure 6:**
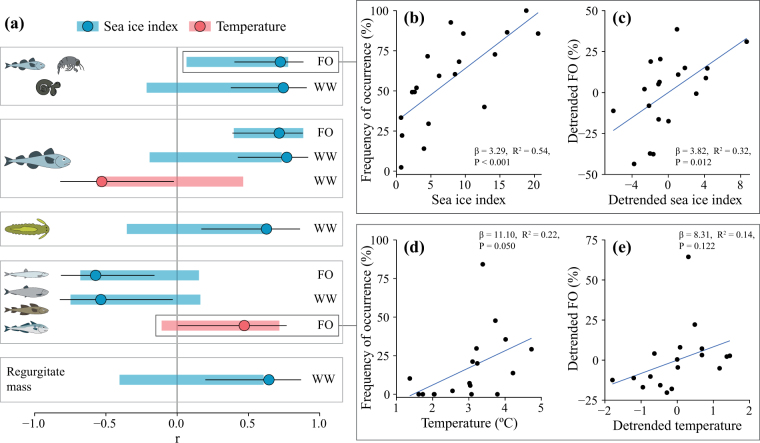
Significant correlations between annual diet indices [frequency of occurrence (FO) and wet weight (WW)] and environmental variables. (**a**) Boxes from the top represent Arctic species, polar cod, *Nereis* spp., Atlantic species, and total regurgitate mass. Circles indicate Pearson’s product moment correlation for non-detrended data, black whiskers 95% confidence intervals (CIs) for the correlations and colored horizontal bars CIs for annually detrended data. If a bar does not cross the grey line at r = 0, the correlation is significant. Colors indicate explanatory variables. (**b**) Linear regression between frequency of occurrence (FO) of Arctic species and sea ice index, (**c**) regression between annually detrended FO of Arctic species and annually detrended sea ice index, (**d**) regression between FO of Atlantic species and temperature, and finally (**e**) regression between annually detrended FO of Atlantic species and detrended temperature. See Extended Data Table 3 for correlation statistics. The icons in the figure were partly made by Dr. Malin Daase.

Frequency of occurrence (FO) and wet weight (WW) of Arctic species in diet, driven by polar cod, showed significant and strong positive correlations with the sea ice index (Fig. 6; see Extended Data Table 3 for correlation statistics). The correlations between FO of Arctic species, polar cod and detrended sea ice index were also significant, indicating that the relationships were not only driven by a common long-term trend. Further, WW of polar cod demonstrated a significant negative correlation with water temperature, although the correlation between detrended variables was not significant.

On the contrary, FO of Atlantic species demonstrated a significant negative correlation with sea ice index, and a positive correlation with temperature in Kongsfjorden. Correlations between detrended variables for Atlantic species were not significant, indicating that the associations were at least partly driven by a common long-term trend. Wet weight of total regurgitate mass and *Nereis* spp. also correlated positively with the sea ice index, but the correlations between detrended variables were not significant.

### Mass of regurgitates and population parameters

The total mass of regurgitates collected from adult kittiwakes decreased significantly from 1997 to 2016 (linear regression, LR: WW (g) = 25.0−0.7 × year, F = 36.5, df = 713, p < 0.001, R^2^ = 0.05). The mass of fish in each sample (i.e. meal mass) was 8.5 g heavier than that for crustaceans (LMM & Tukey: z = −11.1, p < 0.001). The meal mass of fish decreased significantly during the study period (LMM: −0.6 g/year, df = 775, t = −5.0, p < 0.001), whereas crustacean meal mass did not (LMM: df = 343, t = −1.8, p = 0.07). Within fish species, polar cod was the only one to show a linear decrease in meal mass (LMM: −5.5 g/year, df = 877, t = −14.2, p = 0.002).

### Forage fish size

Estimated length of diet fish varied between 22 and 220 mm (Extended Data Fig. 1). Estimated age of foraged fish varied between 0 + and older than 4. The 0 + fish consisted of polar cod (7 in 2007 and 1 in 2011), Atlantic cod (2 in 2006, 6 in 2009, and 3 in 2011), capelin (1 in 2007), and herring (7 in 2013). The earliest records of 0 + Atlantic cod, herring and capelin in diet samples were recorded on July 29, July 5, and July 22, respectively, which implies 100–150 days after spawning period on the coast of mainland Norway^28^.

### Mesopelagic fish

The kittiwake diet samples contained two mesopelagic fishes, glacier lanternfish (*Benthosema glaciale*, Reinhardt 1837) and ribbon barracudina (*Arctozenus risso*, Bonaparte 1840). This was unexpected given the general depth distribution of these fishes (i.e. twilight zone, from 200–1000 m). Another recorded mesopelagic organism was the shrimp crimson pasiphaeid (*Pasiphaea tarda*, Krøyer 1945), that occurred during 11 years and contributed 3.7% of total WW and 6.6% of total FO in the dataset (Table 1, Extended Data Table 1).

## Discussion

Decreasing contribution of Arctic species concomitant with the increase in Atlantic fishes in kittiwake diet samples from Kongsfjorden are likely connected with recent climate warming − driven by increased advection from West Spitsbergen Current and dramatic decrease in marginal sea ice extent around Svalbard^4,29,30^. A strong positive correlation between occurrence of polar cod in kittiwake diet and sea ice cover as well as a similar but opposite correlation for Atlantic water-associated fish species indicate that environmental drivers may be responsible for the observed shifts in diet composition. Such correlations are expected as warmer water masses and less sea ice may disfavor the cold water-adapted polar cod through increased competition from temperate species^31,32^: Sub-Arctic and Atlantic pelagic fishes have higher temperature tolerances and generally benefit from warmer water temperatures^32,33^. Competition from capelin, herring and young Atlantic cod, as well as predation by Atlantic cod and haddock may challenge the sea-ice-adapted polar cod populations forcing their distributions northwards following the retreating polar front and marginal ice zone^32,34^. Consequently, this would lead to decreased abundances of polar cod in the foraging areas of kittiwakes breeding in Kongsfjorden.

Step changes in contributions of Arctic and Atlantic species further indicate that inter-annual variability in the diet samples may be linked to a potential regime-shift in the ecosystem^6,35,36^. The dominance of Arctic species in kittiwake diet suddenly shifted to a more variable diet with high contribution of Atlantic fishes after 2006. In addition, the first occurrence of Atlantic herring in 2013 further increased the contribution of Atlantic species. Winters of 2006 and 2007 were exceptionally warm in Kongsfjorden due to advection of Atlantic water from the West Spitsbergen Current (WSC), changing the hydrography of the fjord for several years^36–38^. The abrupt dominance of capelin in diet composition of kittiwakes and simultaneous decrease in contribution of polar cod appear to be linked to this warming event. The occurrence of herring in diet samples coincided with another warm period with an increased heat content in the WSC, which started in 2012, indicating that the fluctuations of WSC may be behind the changes of Atlantic water-associated fish observed in the kittiwake diet samples^4,26^. The first year when we did not observe polar cod in diet samples was 2015, which coincided with low estimated biomass of polar cod in the Barents Sea due to poor recruitment in 2013–2014^39^, and no polar cod were caught in Kongsfjorden during a trawl survey in 2015^40^. Yet, this does not indicate the disappearance of polar cod from Kongsfjorden, as polar cod recruitment was successful in the Barents Sea in 2015 leading to increased stock sizes^39^, which was also seen in the kittiwake diet samples in 2016.

Fish larvae encountered in diet samples further highlight the role of the WSC as the primary regulator of climate induced ecosystem shifts in the region. The WSC transports pelagic larvae and crustaceans from mainland Norway to the west coast of Svalbard, the minimum transport time being 32–38 days^41^. Barents Sea Atlantic cod, capelin and herring populations spawn along the coast of mainland Norway in February-April^28^. The 0+ Atlantic fish were encountered in kittiwake diet samples 100–150 days after the spawning times, supporting the hypothesis that these fish larvae were advected into the fjord. Marine biological sampling during 1980’s and 1990’s was, at the best, sporadic, and temporal occurrence of Atlantic species in Svalbard fjords could have been overlooked. For example, 0-group herring has been observed in some abundance along the west coast of Svalbard already in 1989^42^, but to our knowledge there are no records that herring had entered west coast fjords until 2006^26,32^. Further, a single capelin was reported in Brünnich’s guillemot diet samples collected from Kongsfjorden as early as 1984^18^. Nevertheless, the change from Arctic-species dominated years, to years with a larger variety of forage species, together with an increasing amount of evidence in the literature e.g.^43^, strongly suggest that west-coast fjord ecosystems in Svalbard are in the state of transition towards Atlantification.

The step change in the diet composition coincided with a step-increase in kittiwake population size (Fig. 5), while breeding success and clutch size appeared to be unaffected by the diet composition. Kittiwakes breeding in Kongsfjorden can find fish by increasing their foraging range or can substitute fish with krill in their diet with little effect on breeding success. Energy content of most common diet items appear relatively similar with *Themisto libellula* being the lowest containing 2.8−4.1 kJ wet g^−1^^44,45^ compared to 4.7−6.8 kJ wet g^−1^ for *Thysanoessa inermis*^44^, 3.5−4.8 kJ wet g^−1^ for capelin^46,47^, and 5.6−6.5 kJ wet g^−1^ for polar cod^45^. Consequently, our results may indicate that, as primarily a boreal species, kittiwakes are adapted to the Atlantification of west coast Svalbard fjords, and possess the ability to replace one diet item with another energy-rich diet item.

Climate driven shifts in the ecosystem structure, echoing to seabird diet composition, have been reported around the globe, including Alaska^15^, the Hudson Bay^16^, the Bering Sea^48^, Newfoundland^49^ and California^50^. Sometimes these shifts are observed as step changes, and may be described as regime-shifts depending on the overall ecosystem effects. We observed a step change in Arctic and Atlantic prey occurrence for kittiwakes breeding in Kongsfjorden, while environmental variables demonstrated fluctuating patterns with linear trends. The shift in prey occurrence may reflect the small geographic scale of the study: the pelagic ecosystem in Kongsfjorden may have responded to the advection events from the WSC^51^, while environmental parameters might be representations of the regional climate that shows fluctuating pattern with a linear trend towards a warmer, more Atlantified, future^52^.

Seabird diet samples provide also other information about ecosystems than changes over time. Occurrence of mesopelagic species in surface-feeding seabird diet appears relatively common and indicates that seabirds routinely have an access to deep-water species: in addition to our study, mesopelagic species have been reported in numerous seabird studies e.g.^15,18,53,54^. Contrary to other studies, kittiwakes collected their diet items during a mid-night sun period and nightly diel vertical migration (DVM) cannot explain the occurrence of mesopelagic species in our study. Instead, zooplankton communities can engage to asynchronous DVM during Arctic summers^55^, and our observations may indicate that also mesopelagic species exercise such vertical migration. Another explanation could arise from upwelling: breeding kittiwakes in Kongsfjorden feed by the tidewater glacier fronts that flow into the fjord^21,56,57^, and mesopelagic fish have been observed near surface by glacier fronts in Alaska^58^. Glacier lanternfish was observed in pelagic trawl catches from approximately 30 m depth in Kongsfjorden, including the inner basin, during summer 2016 (M. Geoffroy & P.E. Renaud, pers. comm.), giving support for the latter hypothesis.

Climate change has already caused noticeable changes in oceanography of high Arctic fjords, but documenting associated ecosystem shifts in such fjords is often challenging due to selectivity of sampling gear, difficulty of access and high costs of ship-based sampling programs. Kittiwakes can act as messengers of the ecosystem state by gathering both fish and macrozooplankton while feeding their chicks, and these data, in turn, can reveal ecosystem-relevant information in an Arctic fjord as demonstrated in this study. The Kongsfjorden system has undergone at least two warming events when large amounts of Atlantic water were advected into the fjord during winters of 2006–2007 and 2012–2013, both of which were associated with increased contribution of Atlantic fish species in the diet. Whether these events were regime-shifts, with no return to the previous cold system with sub-zero temperatures and dominance of Arctic species remain to be seen, although the long-term trends in temperature in the WSC and modelling indicate that further warming is more likely than a cold return^29,59^. However, our findings highlight that the pelagic ecosystem in Kongsfjorden has changed from a cold system towards a warmer, more Atlantic system over the last decade.

## Methods

### Sample collection and handling

Kittiwake diet samples were collected in three colonies in Kongsfjorden, Krykkjefjellet (78°53′46″N, 12°11′43″E), Irgensfjellet (78°59′37″N, 12°7′46″E), and Observasjonsholmen (78°56′20″N, 12°17′5″E), Svalbard (Fig. 1b) during incubation (June to mid-July) and chick-rearing (mid-July to August) periods. Diet material was collected during 1997–98 and 2004–2016 in connection with other studies involving capture and handling of adults and chicks. Kittiwakes often regurgitate contents of their proventriculus during handling^17^. Regurgitates were collected into marked plastic bags and frozen at -18 °C. In the lab, prey items were identified to lowest possible taxon, using a variety of identification literature^60,61^. Fish species were determined either from body or otolith morphology when whole fish were too digested to be recognizable^62,63^. Wet biomass (WW) of each taxon was measured. Diet items were categorized for statistical analyses, first allocating them to the main groups “fish”, “Crustacea” and “other”, then to meaningful groups or subgroups after their origin (Table 1).

Out of the 879 collected kittiwake diet samples, 142 were obtained during incubation and 737 during chick rearing. Diet samples were from both adults (715) and chicks (164). Adult samples were collected from males (229) and females (202). The sex was unknown in 284 cases. Most samples were taken at Krykkjefjellet (392), followed by Irgensfjellet with 343 samples, but in 144 cases sampling site was not recorded.

Otoliths found in diet samples were counted and measured to the nearest 0.1 mm (length and width) with a calibrated stereomicroscope. Length of otolith pairs that likely originated from a same fish was averaged. Length of polar cod, capelin, herring, Atlantic cod and glacier lanternfish was estimated from otolith lengths using linear regression models (Extended Data Table 4). Studies reporting the otolith size – fish length regressions used frozen material, which could have reduced the estimates by 1–4% from actual fish lengths^64^. Age-groups were further approximated using the estimated lengths and available literature (Extended Data Table 4).

Additional diet data covering years 1982–1984 and 1987^18,65^ were added to the dataset. These data contained only binary presence/absence information and therefore did not allow mass-based analyses.

### Diet indices

Four different metrics were used to describe the kittiwake diet data for each year: (1) Frequency of occurrence [FO (%)], calculated as a proportion of samples containing a given diet item, was used to combine 1982–1987 binary data with 1997–2016 mass-based data. (2) Average wet weight [WW (g)] per sample of the different prey species was used as a proxy of energetic importance of diet items collected during 1997–2016 when related statistical analyses were able to handle outlier-heavy non-normal data. (3) Average mass percentage (AP), calculated as the mean of the relative contribution of a given prey item to the total mass of samples was used with mass data based statistical tests that were not able to handle non-normal data. Average mass percentage reduces the influence of outliers that are often associated with compositional diet analyses. It gives equal weight to each sample in the analysis and allows the use of uncertainty statistics for the mean estimate unlike frequency of occurrence^66^. Finally, (4) Average meal mass [MM (g)], calculated as the mean wet weight of a given prey species in all samples excluding zeros, was used to explain trends in wet weight data. This metric is the mean mass of a given prey item in samples where the item was present. Frequency of occurrence and AP demonstrated strong correlation with each other (r = 0.99), whereas WW correlated weakly [r = 0.48 (AP) and 0.47 (FO)] with these indices.

### Environmental data

A set of simple climate indices was selected to potentially explain variation in diet composition of kittiwakes. Following Prop *et al*.^3^, data on daily sea ice concentration were downloaded from National Snow and Ice Data Center, University of Colorado (ftp://sidads.colorado.edu/pub/DATASETS/nsidc0051_gsfc_nasateam_seaice/final-gsfc/north/daily/) for four adjacent 25×25 km blocks outside Kongsfjorden. Monthly average concentrations (January–December) were further averaged to annual values for the period 1980–2016. These data represent large-scale sea ice conditions at Northwest Spitsbergen. Fine-scale data on fast ice concentration within Kongsfjorden are available for the time-period 2004–2015^4^ and strongly correlate with the large-scale sea ice data (r = 0.89, p < 0.001). Hence, we believe our large-scale sea ice index also reflect the annual variation in local fast ice conditions within Kongsfjorden for the study period.

Water temperature data for Kongsfjorden was obtained from Tverberg *et al*.^4^ and was calculated from ship-based CTD casts taken at three stations with bottom depth >100 m within the fjord in July–September. The values are volume-weighted mean temperatures, where the value for each depth level is multiplied by the area of Kongsfjorden at that depth level, and the sum of resulting values is divided by the total volume of the fjord. The authors claim that the method represents total heat content in the fjord better than simple averaging methods.

### Demographic data

Breeding success and clutch size estimates were obtained for the colonies at Krykkjefjellet and Irgensfjellet (Fig. 1b). Breeding success index was calculated as the number of chicks >12.5 days old per active nest, and clutch size was calculated as number of eggs per active nest^57,67^. Population size estimates were collected from the Ossian Sarsfjellet colony (Fig. 1b) during 1988–2016, resulting in a total of 27 colony-size estimates (no estimate for 1989 and 2009). For each year between mid- and end of June (incubation period), the number of active nests (i.e. nest with an incubating kittiwake) were counted in four different plots. All activities including colony monitoring, bird captures, handling, and food sampling were approved and conducted according to the permits provided by the Governor of Svalbard and by the Norwegian Animal Research Authority.

### Statistical methods

Effects of breeding stage (incubating/rearing), bird age (adult/chick), and breeding colony (Blomstrand/Krykkjefjellet / not recorded) on composition of binary kittiwake diet data (Arctic species, polar cod, *T*. *libellula*, Atlantic species, capelin and *T*. *inermis*) were analyzed using general linear mixed effect models (GLMM), logistic functions and year as a random effect (see Text S2 for details). Similar effects on total regurgitate mass were analyzed using linear mixed effect models (LMM) and logarithm-transformed mass-based data for 1997–2016. Breeding stage and bird age significantly affected diet composition, but GLMMs including breeding stage and bird age echoed the conclusions from simple correlations and linear regressions (LR) using annual averages (Text S2). Consequently, annual frequency of occurrence and average wet weight of diet species were correlated with environmental variables (temperature and sea ice index) and with population parameters using linear regressions, two-sided Pearson product moment correlations, and both time-detrended as well as non-detrended values for dependent and independent variables.

The wet weight of Arctic and Atlantic species in diet samples each year was compared using two-sample exact permutation tests estimated by Monte Carlo and two-sided null-hypothesis^68^. Years with similar prey composition (FO) were grouped using hierarchical clustering (*hclust* in^69^) with Ward’s clustering criterion (“ward.D2”) on Bray-Curtis dissimilarity matrix (*vegdist* in^70^). The dissimilarity matrix was further plotted using nonmetric multidimensional scaling (nMDS, *metaMDS* in^70^).

Step changes in FO time-series for Arctic and Atlantic species, as well as for explanatory variables (sea ice index, temperature, population size, clutch size and breeding success) were identified using change-point analysis^71^ and a model selection routine (see Text S1 for details). Since the focus was on identifying possible step changes in the diet data, explanatory variables were constrained to exclude changes before 1997. Change-points are reported as the last year of the previous regime.

All statistics were run using the R statistical programming environment^69^. The maps presented in this article were made using Ocean Data View^72^. Depth data for Kongsfjorden were obtained from Kartverket (http://www.kartverket.no/).

### Biases and assumptions

Analysis of diet material introduces a bias to our results since soft-bodied organisms are more easily digested than bone-structures of fish^73^. This could have decreased the contribution of crustaceans and *Nereis* spp. throughout the study. Further, the contribution of unidentified fish was large (up to 60% FO and 40% AP) during some years and could have interfered with the reported patterns of polar cod and Atlantic fish. These species were relatively similar in size (Extended Data Fig. 1), and assimilation efficiency related to polar cod and capelin only differ by ~10% in kittiwakes^74^. It was therefore assumed that polar cod and Atlantic fish species were digested at similar rates, and further, that unidentified fish were randomly distributed among these fish categories. Consequently, unidentified fish were not considered in the statistical analyses.

Observations and GPS tracking data suggest that the kittiwakes were likely to forage within Kongsfjorden during the time of sampling: 50−85% of trips in studied males and 98–100% of trips in females breeding in Krykkjefjellet were located inside Kongsfjorden before egg-laying in 2008–2010 ^57^. Further, GPS tracking conducted at several kittiwake colonies in Kongsfjorden in the periods 2008–2009 and 2014–2016 indicates that foraging mostly takes place within the fjord during incubation and chick rearing^19,20^. Since our diet data were collected from incubating and chick-rearing birds, most kittiwakes were likely to forage within or adjacent to Kongsfjorden. Nevertheless, we cannot exclude the possibility that kittiwakes forage outside the fjord, for example during a year when prey abundance was low within the fjord.

### Data availability

The datasets are openly available in the Norwegian Polar Data Centre (10.21334/npolar.2017.26dbd004).

## Electronic supplementary material

Supplementary information
