# Supplementary material for: Black-legged kittiwakes as messengers of Atlantification in the Arctic
Source: Sci Rep. 2018 Jan 19;8:1178. doi: 10.1038/s41598-017-19118-8 (PMC5775339; doi:10.1038/s41598-017-19118-8)
Supplement: Supplementary file 1 — Supplementary information [file 41598_2017_19118_MOESM1_ESM.pdf]

Supplementary information and extended data for

Black-legged kittiwakes as messengers of Atlantification in the Arctic

Authors

Mikko Vihtakari, Jorg Welcker, Børge Moe, Olivier Chastel, Sabrina Tartu, Haakon Hop, Claus Bech, Sébastien Descamps, Geir Wing Gabrielsen

Scientific Reports (2018). DOI: 10.1038/s41598-017-19118-8

Extended Data

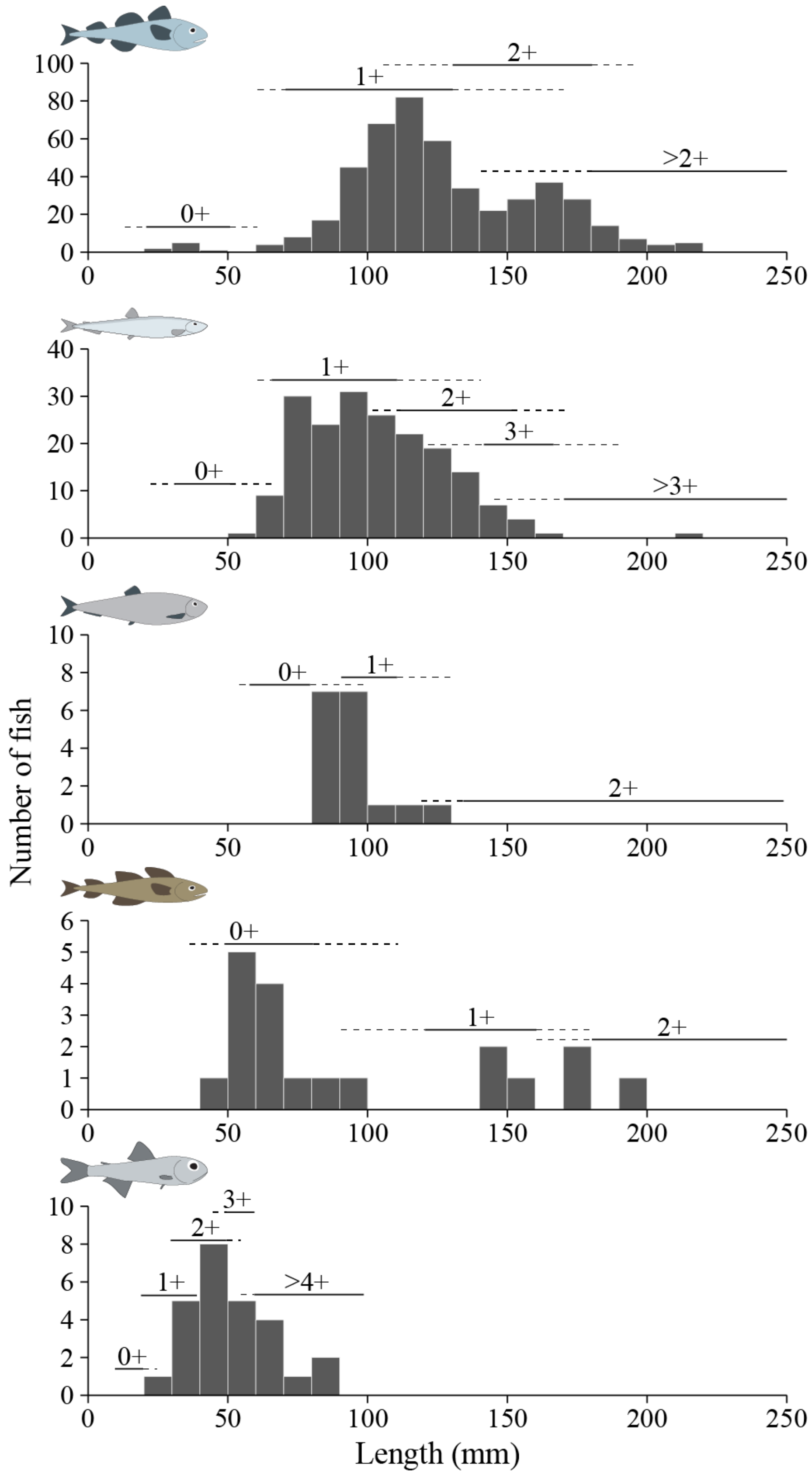

Extended Data Figure 1. Length-frequency histograms of polar cod, capelin, herring, Atlantic cod, and glacier lanternfish estimated from otolith lengths. Histograms include forage fish from all available years (2004–2016). Each bar comprises 10 mm in length. Polar and Atlantic cod are given as FL, capelin and Atlantic herring as TL, whereas glacier lanternfish as SL. Lines above histograms indicate typical size range for age groups of forage fish in the Barents Sea or the Norwegian coast. Solid line indicates range for average lengths, whereas dashed lines illustrate range including 2 × standard deviation. See Extended Data Table 4 for otolith size – fish-length regression equations and sources. The icons in the figure were partly made by Dr. Malin Daase.

Extended Data Table 1. Composition of kittiwake regurgitates from Kongsfjorden in 1997-2016 as average mass percentages (AP± SE). Number of samples is given under the label for each year.

	**1997**	**1998**	**2004**	**2005**	**2006**	**2007**	**2008**	**2009**	**2010**	**2011**	**2012**	**2013**	**2014**	**2015**	**2016**
**Diet item**	28	11	37	136	102	57	52	64	88	53	75	65	24	42	45
Fish	67.5 (8.7)	64.3 (14.5)	86.3 (5.1)	94.9 (1.6)	66.0 (4.2)	97.0 (2.1)	59.6 (6.6)	79.3 (4.7)	41.0 (5.0)	78.3 (5.0)	80.4 (4.2)	84.4 (4.2)	57.2 (9.9)	17.1 (5.7)	91.6 (3.3)
Polar cod	67.4 (8.7)	64.3 (14.5)	76.7 (6.4)	87.7 (2.6)	54.3 (4.4)	12.3 (4.2)	24.8 (5.8)	47.1 (5.7)	16.0 (3.7)	48.9 (6.7)	31.3 (5.2)	28.5 (5.2)	15.3 (6.8)	-	14.6 (4.8)
Atlantic fish	-	-	-	1.5 (1.0)	2.2 (1.4)	79.9 (5.0)	12.6 (4.0)	18.8 (4.2)	9.2 (3.0)	3.2 (2.1)	14.0 (3.8)	34.9 (5.5)	22.0 (7.8)	7.1 (4.0)	29.6 (6.5)
Capelin		-	-	1.5 (1.0)	0.1 (0.1)	79.9 (5.0)	12.6 (4.0)	16.3 (4.1)	9.2 (3.0)	1.2 (1.2)	11.5 (3.5)	1.4 (1.4)	-	-	10.2 (4.2)
Atlantic herring	-	-	-	-	-	-	-	-	-	-	-	33.3 (5.5)	6.1 (3.9)	7.1 (4.0)	19.4 (5.8)
Atlantic cod	-	-	-	-	0.9 (0.7)	-	-	2.5 (1.4)	-	1.3 (0.9)	1.3 (1.3)	0.3 (0.3)	15.9 (7.4)	-	-
Haddock	-	-	-	-	1.2 (1.0)	-	-	-	-	0.6 (0.6)	1.2 (1.2)	-	-	-	-
Other fish	-	-	6.9 (3.6)	5.7 (1.8)	8.3 (2.4)	-	0.5 (0.5)	3.1 (1.8)	2.9 (1.6)	6.2 (3.0)	6.5 (2.8)	0.7 (0.5)	0.5 (0.5)	2.8 (2.1)	7.0 (3.5)
Glacier lanternfish	-	-	6.2 (3.6)	4.5 (1.6)	5.9 (2.0)	-	-	-	1.6 (1.2)	3.2 (2.2)	1.2 (1.2)	0.0 (0.0)	-	1.9 (1.9)	3.7 (2.6)
White barracudina	-	-	-	1.1 (0.8)	1.9 (1.3)	-	0.5 (0.5)	1.3 (0.9)	1.3 (0.9)	0.9 (0.9)	3.2 (1.9)	-	-	-	2.2 (2.2)
Daubed shanny	-	-	0.7 (0.7)	0.1 (0.1)	0.1 (0.1)	-	-	1.8 (1.6)	-	0.2 (0.2)	-	0.4 (0.4)	-	-	1.1 (1.1)
Snakeblenny	-	-	-	-	0.4 (0.4)	-	-	-	-	-	-	0.3 (0.3)	0.5 (0.5)	-	-
Unidentified fish	0.1 (0.1)	-	2.7 (2.7)	-	1.3 (1.0)	4.8 (2.6)	21.6 (5.6)	10.2 (3.7)	12.9 (3.5)	20.0 (5.0)	28.6 (5.1)	20.3 (4.9)	19.3 (8.0)	7.1 (4.0)	40.4 (7.1)
Crustacea	17.9 (6.9)	33.9 (14.0)	7.1 (3.8)	1.1 (0.6)	19.3 (3.6)	2.6 (1.8)	38.3 (6.5)	16.3 (4.3)	55.5 (5.1)	13.2 (4.1)	17.4 (4.0)	15.3 (4.2)	42.6 (9.9)	82.9 (5.7)	8.4 (3.3)
Krill (*Thysanoessa*)	13.9 (6.1)	33.9 (14.0)	3.8 (2.8)	0.9 (0.6)	11.4 (3.0)	2.6 (1.8)	21.6 (5.4)	13.7 (4.1)	52.4 (5.1)	12.8 (4.0)	10.2 (3.3)	8.0 (3.3)	42.4 (9.9)	77.7 (6.4)	5.4 (3.1)
T. inermis	13.9 (6.1)	33.9 (14.0)	3.8 (2.8)	0.9 (0.6)	10.4 (2.9)	2.6 (1.8)	21.4 (5.4)	13.7 (4.1)	52.4 (5.1)	12.8 (4.0)	10.2 (3.3)	8.0 (3.3)	42.4 (9.9)	77.7 (6.4)	5.4 (3.1)
T. longicaudata	-	-	-	-	1.0 (1.0)	-	0.2 (0.2)	-	-	-	-	-	-	-	-
Themisto	4.0 (3.6)	-	3.1 (2.7)	0.1 (0.1)	6.7 (2.2)	-	16.6 (4.9)	2.4 (1.7)	3.0 (1.6)	0.1 (0.1)	5.2 (2.4)	6.7 (2.9)	0.1 (0.1)	0.1 (0.1)	0.1 (0.1)
T. libellula	4.0 (3.6)	-	3.1 (2.7)	0.1 (0.1)	2.8 (1.3)	-	16.6 (4.9)	2.4 (1.7)	3.0 (1.6)	0.1 (0.1)	4.5 (2.3)	6.7 (2.9)	0.1 (0.1)	0.1 (0.1)	0.1 (0.1)
T. abyssorum	-	-	-	-	3.9 (1.8)	-	-	-	-	-	0.7 (0.6)	-	-	-	-
Shrimp	-	-	0.2 (0.2)	0.1 (0.1)	-	-	0.2 (0.1)	0.0 (0.0)	-	0.3 (0.2)	1.8 (1.3)	0.2 (0.1)	-	0.5 (0.4)	2.5 (1.4)
Pandalus borealis	-	-	0.2 (0.2)	0.1 (0.1)	-	-	0.2 (0.1)	0.0 (0.0)	-	-	-	0.2 (0.1)	-	0.5 (0.4)	2.5 (1.4)
Pasiphaea tarda	-	-	-	-	-	-	-	-	-	0.3 (0.2)	1.8 (1.3)	-	-	-	-
Unidentified Crustacea	-	-	-	-	1.2 (0.5)	-	-	0.2 (0.2)	0.1 (0.1)	-	0.1 (0.1)	0.5 (0.4)	-	4.8 (3.3)	0.4 (0.3)
Other	14.6 (6.7)	1.8 (1.8)	6.7 (3.7)	3.9 (1.5)	14.6 (3.1)	0.4 (0.4)	2.1 (1.9)	4.4 (2.3)	3.5 (1.8)	8.5 (3.5)	2.2 (1.5)	0.3 (0.2)	0.2 (0.2)	-	-
Limacina helicina	10.7 (6.0)	-	-	0.5 (0.5)	1.3 (1.0)	-	-	-	-	3.2 (2.3)	0.5 (0.5)	-	-	-	-
Nereis spp.	3.9 (3.6)	1.8 (1.8)	6.7 (3.7)	1.6 (0.9)	11.9 (2.9)	0.4 (0.4)	2.1 (1.9)	4.4 (2.3)	3.5 (1.8)	5.1 (2.8)	0.4 (0.2)	0.3 (0.2)	0.2 (0.2)	-	-
Parasagitta elegans	-	-	-	1.8 (1.1)	-	-	-	-	-	-	1.3 (1.3)	-	-	-	-

Extended Data Table 2. Two-sample permutation test results to compare wet weight contribution of Arctic and Atlantic species (See Table 2 for species grouping and Fig. 5 for summary). Columns from the left: Year; mean (x̄) and bootstrapped 95% confidence intervals for the mean for Arctic species; Similar values for Atlantic species; Difference in wet weight (ΔWW) and the P value using exact two-sided permutation test by Monte Carlo; "Arctic", the number of samples where Arctic taxa was present in a given year and "Atlantic" similar number for Atlantic taxa. Bold *p* values are significant.

	Arctic species			Atlantic species			Permutation test		Occurrence	
Year	x̄	Min	Max	x̄	Min	Max	ΔWW	P	Arctic	Atlantic
1997	20.2	12.5	27.1	0	0	0	20.2	**0.00**	24	0
1998	17.1	4.3	28.1	0	0	0	17.1	**0.01**	6	0
2004	12.3	9.1	15.4	0	0	0	12.3	**0.00**	26	0
2005	21.4	18.6	24.3	0.3	-0.1	0.5	21.1	**0.00**	118	3
2006	10.1	6.6	13.1	0.8	-0.3	1.5	9.3	**0.00**	40	10
2007	2.4	0.4	3.9	12.7	9.7	15.4	-10.3	**0.00**	8	46
2008	3.3	2.1	4.6	3.7	0.9	6.1	-0.4	0.84	20	10
2009	7.4	5.2	9.4	4.9	1.9	7.5	2.5	0.20	37	19
2010	3.7	0.6	5.9	3.3	0.7	5.7	0.4	0.92	15	7
2011	12.4	7.9	16.6	0.4	-0.2	0.8	12	**0.00**	27	2
2012	7.7	3.3	11.4	2	0.5	3.2	5.7	**0.00**	25	12
2013	5.9	2.7	8.5	6.9	4	9.5	-1	0.64	28	28
2014	2.2	0	4	5.8	0.8	9.9	-3.6	0.19	8	7
2015	0	0	0	1.2	-0.4	2.4	-1.2	0.26	1	3
2016	3	-0.1	5.5	6.1	2.7	9.3	-3.1	0.19	6	10

Extended Data Table 3. Correlation statistics between diet indices and explanatory variables (See Fig. 6 for summary). "Index" column indicates the corresponding diet index, "a" the intercept for linear regression using non-detrended values, "b" the slope, "N" the number of annual averages, "P" the *p* value, "R^2" the coefficient of determination and "r" the Pearson correlation. Bold *p* values are significant.

			Non-detrended						Detrended			
Diet item	Variable	Index	a	b	N	P	R^2	r	b	P	R^2	r
Arctic species	Sea ice index	FO	31.6	3.3	19	**0.000**	0.53	0.73	3.4	**0.027**	0.26	0.51
Arctic species	Sea ice index	WW	2.5	1.1	15	**0.001**	0.56	0.75	0.5	0.221	0.11	0.34
Arctic species	Temperature	FO	91.3	-11.8	18	0.109	0.15	-0.39	-6.7	0.303	0.07	-0.26
Arctic species	Temperature	WW	19.0	-3.4	15	0.067	0.24	-0.49	-0.1	0.959	0.00	-0.01
Arctic species	Population size	FO	100.9	-0.4	14	0.237	0.12	-0.34	-0.1	0.701	0.01	-0.11
Arctic species	Population size	WW	19.0	-0.1	14	0.321	0.08	-0.29	0.0	0.940	0.00	-0.02
Polar cod	Sea ice index	FO	20.8	3.3	19	**0.001**	0.51	0.72	5.6	**0.000**	0.52	0.72
Polar cod	Sea ice index	WW	1.6	1.1	15	**0.001**	0.59	0.77	0.5	0.194	0.13	0.36
Polar cod	Temperature	FO	88.7	-13.7	18	0.069	0.19	-0.44	-9.2	0.188	0.11	-0.33
Polar cod	Temperature	WW	19.3	-3.8	15	**0.043**	0.28	-0.53	-0.3	0.822	0.00	-0.06
Polar cod	Population size	FO	111.8	-0.6	14	0.096	0.21	-0.46	-0.3	0.374	0.07	-0.26
Polar cod	Population size	WW	18.6	-0.1	14	0.310	0.09	-0.29	0.0	0.961	0.00	-0.01
Atlantic species	Sea ice index	FO	31.8	-2.0	19	**0.011**	0.33	-0.57	-1.8	0.177	0.10	-0.32
Atlantic species	Sea ice index	WW	5.5	-0.4	15	**0.040**	0.29	-0.53	-0.4	0.162	0.15	-0.38
Atlantic species	Temperature	FO	-16.2	11.1	18	**0.048**	0.22	0.47	8.3	0.122	0.14	0.38
Atlantic species	Temperature	WW	-2.1	1.7	15	0.079	0.22	0.47	1.3	0.274	0.09	0.30
Atlantic species	Population size	FO	-18.7	0.3	14	0.268	0.10	0.32	0.2	0.461	0.05	0.22
Atlantic species	Population size	WW	-3.2	0.1	14	0.247	0.11	0.33	0.0	0.447	0.05	0.22
Total mass	Sea ice index	WW	11.3	0.8	15	**0.009**	0.42	0.65	0.2	0.624	0.02	0.14
Total mass	Temperature	WW	25.0	-3.0	15	0.084	0.21	-0.46	-0.1	0.973	0.00	-0.01
Total mass	Population size	WW	21.8	-0.1	14	0.546	0.03	-0.18	0.0	0.648	0.02	0.13

Extended Data Table 4. Linear regressions used to estimate fish lengths from otolith size together with references used to estimate age-groups of forage fish (Extended Data Figure 1). Regression column represents the linear regression used (FL = fork length, TL = total length, SL = standard length, OL = otolith length and OW = otolith width), with range column indicating the length range (mm) of fish used for the linear models given in Source column. References for age-group length estimates are given in the Age source column.

Species	Regression	Range	Source	Age source
Polar cod	$FL = 24.71 \times OL + 6.09$	33–292	1	2–6
Capelin	$TL = 56.22 \times OL + 11.73$	48–204	1	6–9
Atlantic herring	$TL = 57.86 \times OL + 15.63$	66–190	1	6, 8–10
Atlantic cod	$FL = 22.55 \times OL - 15.00$	13–293	1	8–11
Glacier lanternfish	$SL = 31.79 \times OW - 3.31$	37–85	12	13

Supporting information

Text S1. Change-point analysis

Methods

The change-point analysis was conducted using frequency of occurrence data for Arctic and Atlantic species, as well as for explanatory variables (sea ice index, temperature, population size, clutch size and breeding success). Since the focus was on identifying possible step changes in the diet data, explanatory variables were constrained to exclude changes before 1997. Change-points are reported as the last year of the previous regime.

One step change for each dataset was identified using the *cpt.mean* function from the *changepoint* package [14] using the "AMOC" (At Most One Change) method [15]. The p-value for change-points was estimated using the maximum log likelihood method and was inverted to acquire a p-value, which interpretation is analogous to classical null-hypothesis testing [14,15].

The change-point identifying routine assumed two constant mean values (i.e. regressions without slope), yet our data demonstrated linearly increasing or decreasing patterns. Therefore, to further examine the direction of changes, the identified years for step changes in the dataset were compared to alternative linear models using piecewise regressions and model selection. First, a selection of feasible models was formulated: 1) linear regression covering the entire dataset ($y = a \times t + b$, where *y* was the response variable, *t* years from 1982 onwards, *a* the slope, and *b* the intercept). This model represented a gradual long-term change in a time-series. 2) Constant mean value covering the entire dataset ($y = b$). The model emulated no change in a time-series. 3) Constant mean values with a change-point *m* identified by the change-point analysis ($y_{n-m} = b_1, y_{(m+1)-2016} = b_2$), where *n* was 1982

for diet data and 1997 for explanatory variables. This model was identified by the change-point analysis and emulated a situation with a sudden step change in a time-series. 4) Two linear regressions with a constant slope (i.e. no interaction term) and a change-point ($y_{n-m} = a \times t + b_1$; $y_{(m+1)-2016} = a \times t + b_2$). These models emulated a situation with a gradual change of a similar direction complemented by a step change. Finally, 5) two linear regressions with a free slope (i.e. with an interaction term) and a change-point ($y_{n-m} = a_1 \times t + b_1$; $y_{(m+1)-2016} = a_2 \times t + b_2$). This model imitated a situation where the time series demonstrated two different gradually changing regimes throughout years. Models for each variable were then compared and best fitting one was chosen using Akaike's Information Criterion (AIC). Only models 1 and 2 were used for explanatory variables that did not demonstrate a step change according to the change-point analysis.

Results

The change-point analysis indicated a shift in diet data and population size in 2006 (Table S1). Similar change-point was identified for sea ice index in 2005, whereas temperature, clutch size and breeding success data demonstrated no significant change-points.

Table S1. Change-points in analyzed kittiwake diet- and explanatory variable time-series as identified by the change-point analysis.

Variable	Change-point	P value
Arctic species	2006	>0.001
Atlantic species	2006	>0.001
Sea ice index	2005	0.006
Temperature	None	
Population size	2006	>0.001
Clutch size	None	
Breeding success	None	

Model selection confirmed the step change in Arctic and Atlantic species, but indicated that a linear model described sea ice index time-series better than piecewise regressions (Table S2). Nevertheless, the difference in residual standard error between models with a change-point was only 0.1-0.2 suggesting no large differences in which model described the data best. Model selection further indicated a regular increasing trend for temperature from 1997 until 2016 (0.1 degrees / year, $F = 12.6$, $p = 0.002$) and no significant change for clutch size and breeding success over time. Population size was best described by a piecewise regression with a degreasing trend (-6.8 nesting pairs / year, $F = 5.6$, $p = 0.008$) until 2006 and an increasing trend (1.4 nesting pairs / year) thereafter.

Table S2. Model selection based on the change-point analysis. Columns from the left: “Variable” specifies the fitted response variable, “Model” the used model, “Np” number of model parameters, “Df” degrees of freedom, σ the residual standard error, AIC the Akaike’s Information Criterion and ΔAIC the difference between model with lowest AIC and corresponding model. Best fitting models, as indicated by lowest AIC, are highlighted using bold font.

Variable	Model	Np	Df	σ	AIC	ΔAIC
Arctic species	Linear 1982-2016	2	17	23.5	177.8	8.3
	Constant 1982-2016	1	18	27.9	183.4	13.9
	**Constant change-point 2006**	**2**	**17**	**18.9**	**169.5**	**0**
	Linear no interaction change-point 2006	3	16	19.5	171.5	2
	Linear interaction change-point 2006	4	15	19.3	172	2.4
Atlantic species	Linear 1982-2016	2	17	19.5	170.8	4.5
	Constant 1982-2016	1	18	21.9	174.2	7.9
	**Constant change-point 2006**	**2**	**17**	**17.4**	**166.3**	**0**
	Linear no interaction change-point 2006	3	16	17.9	168.3	2
	Linear interaction change-point 2006	4	15	17.4	168.1	1.8
Sea ice index	**Linear 1997-2016**	**2**	**18**	**3.0**	**105.2**	**0**
	Constant 1997-2016	1	19	4.3	117.9	12.6
	Constant change-point 2005	2	18	3.2	106.6	1.3
	Linear no interaction change-point 2005	3	17	3.1	106.3	1
	Linear interaction change-point 2005	4	16	3.1	107.9	2.6
Temperature	**Linear 1997-2016**	**2**	**18**	**0.7**	**47.3**	**0**
	Constant 1997-2016	1	19	0.9	55.9	8.6
Population size	Linear 1997-2016	2	17	27.1	183.2	9
	Constant 1997-2016	1	18	27.3	182.5	8.3
	Constant change-point 2006	2	17	24.7	179.6	5.4
	Linear no interaction change-point 2006	3	16	23.7	179	4.8
	**Linear interaction change-point 2006**	**4**	**15**	**20.5**	**174.2**	**0**
Clutch size	Linear 1997-2015	2	16	0.2	-9.9	1.2
	**Constant 1997-2015**	**1**	**17**	**0.2**	**-11.1**	**0**
Breeding success	Linear 1997-2014	2	14	0.4	18.8	1.8
	**Constant 1997-2014**	**1**	**15**	**0.4**	**17**	**0**

Text S2. Environmental correlations using binary data and rationale behind keeping data

Background

Breeding stage and bird age significantly affected diet composition of the kittiwakes (Table S3). Removal of incubating birds would have increased frequency of occurrence (FO) of Atlantic species and decreased the contribution of Arctic species especially in 2009, but also in 2007-2008 (Table S4). Further removal of chicks would have enhanced this effect in 2008, and increased FO of both Atlantic and Arctic species in 2016 (Table S5). Removal of samples, however, would have considerably reduced sample size, and consequently the reliability of our dataset. Further, the removal would not have influenced our conclusions about change-points in the dataset nor about the environmental correlations. Finally, we did not feel confident in removing data purely based on statistics: the focus of this article was to look at ecosystem changes in the foraging area of kittiwakes breeding in Kongsfjorden. Bird age or breeding stage should not affect the interpretation of the ecosystem. On the contrary, a wider dataset can be seen as a benefit for such application. Consequently, diet data from incubating birds and chicks were not removed. In this SI, we show that keeping the incubating birds and chicks in the dataset did not influence the results, and further that the simplified approach of using FOs instead of raw data with mixed models did not affect our conclusions.

Methods

Effects of breeding stage (incubating / rearing), bird age (adult / chick), and breeding colony (Blomstrand / Krykkjefjellet / Not recorded) on composition of binary kittiwake diet data (Arctic species, polar cod, *T. libellula,* Atlantic species, capelin and *T. inermis*) were analyzed using general linear mixed effect models (GLMM), logistic functions and year as a random effect with the *lme4* package [16] for R [17]. The GLMM models were formulated as

follows: $glmer(value \sim x + (1|year) - 1, data = y, family = binomial)$, where $value$ was the binary column of diet data for 1982-2016, x the factor column specifying breeding stage, bird age or colony, and y the data frame. Similar effects on total regurgitate mass were analyzed using linear mixed effect models (LMM) and logarithm-transformed mass-based data for 1997-2016. The LMM models were formulated as $lmer(\log(value) \sim x + (1|year) - 1, data = y)$. Significance of these models was tested by comparing the models to a null model with removed x in the model formulation using Chi-square tests [18]. The significance was confirmed by estimating confidence intervals for model parameters using likelihood profile estimation.

Effects of breeding stage and bird age on environmental correlations (see Fig. 6) was examined by, both, GLMMs on binary composition 1982-2016 data and LMMs on logarithm-transformed mass-based 1997-2016 data. The models were constructed by using year as a random effect intercept and both stage and age as random effect slopes for each year. The resulting model for binary data was formulated as $glmer(value \sim z + (stage + age|year), data = y, family = binomial)$, where $value$ was the binary column of diet data (Arctic species, polar cod or Atlantic species) and z the numeric column of an environmental factor (sea ice index, temperature or population size). Marginal and conditional R^2 values were calculated from the models using the *MuMIn* package [19] and used to access how well models described the underlying data [20].

Results

Wet weight was significantly affected by breeding stage with total regurgitate mass being 41 % heavier during chick rearing compared to incubation (Table S3). Further, samples collected from adults were on average 46% heavier compared to chicks. These total

regurgitate mass differences were, however, not critical to overall conclusions in this paper, as we examined mostly the composition of diet samples.

Breeding stage and bird age significantly affected diet composition of the kittiwakes, whereas breeding colony did not (Table S3). Atlantic species were, in general, more common during chick-rearing and polar cod during incubation. These significant differences were, however, caused by a few years. Removal of incubating birds would have led to removal of 1984 samples altogether reducing contribution of Arctic species (Table S4). Further, the year 2009 would have scored a lower FO on Arctic species and a higher score on Atlantic species. Also 2008 and 2007 would have been affected. Removal of chicks would have further decreased the contribution of Arctic species in 2010 and increased the contribution of Atlantic species in 2008. These years occurred after 2006, which was identified as the change-point in our dataset. Consequently, removal of samples would have only enhanced already significant change-point analysis and, therefore, we decided to keep the samples.

Mixed effect models with age and stage as random slopes and year as random intercept led to similar results than linear regressions with annual FO and WW values (Extended Data Table 3, Table S6). Both, binary and mass-based data on Arctic species significantly correlated with sea ice index (Table S6). This correlation was caused by polar cod. Further, binary data for Atlantic species correlated negatively with sea ice index and positively with temperature. Finally, total regurgitate mass correlated positively with sea ice index. It should be noted that while mixed effect models use the entire raw dataset and may illustrate the patterns in a dataset more precisely, they are also subject to more pitfalls than simple linear regressions using average values due to their complexity and larger dataset [21]. Mixed effect models may be “a more correct” way of describing patterns in the dataset, but their interpretation is also more difficult than the interpretation of correlations. Therefore we

believe that the correlations and linear regressions in the main part of the article appeal to a wider audience than mixed effect models. Consequently, given the similar results from both methods, we opted for the simpler method in the main text of the article.

Table S3. Effects of breeding stage (“Stage”), bird age (“Age”) and breeding colony (“Colony”) on composition and total regurgitate mass of diet samples. Annual composition of samples were compared using binary absence/presence data for 1982-2016 and general linear mixed effect models (GLMM), whereas logarithm-transformed wet weight data for 1997-2016 and linear mixed effect models (LMM) were used for total regurgitate mass. Comparisons were conducted using Chi-square tests (χ^2). Parameter estimates (β), number of samples (N) and profile likelihood confidence intervals for β (Min and Max) are given for each factor level. R^2_m and R^2_c columns give marginal and conditional R^2 values, respectively. Bold *p*-values are significant.

		Term 1 (intercept)					Terms 2 and 3 (slope)					Statistics			
Item	Variable	Term	N	β	Min	Max	Term	N	β	Min	Max	χ^2	P	R^2_m	R^2_c
Arctic species	Stage	Incubation	147	0.61	0.41	0.78	Rearing	773	0.58	0.39	0.74	0.34	0.561	0.00	0.39
	Age	Adult	756	0.57	0.39	0.74	Chick	164	0.64	0.44	0.80	1.68	0.195	0.00	0.39
	Colony	Blomstrand	343	0.56	0.37	0.74	Krykkjefjellet	392	0.59	0.40	0.76	0.43	0.806	0.00	0.38
	Colony						Not recorded	185	0.60	0.40	0.77				
Polar cod	Stage	Incubation	147	0.54	0.34	0.74	Rearing	773	0.42	0.25	0.61	4.29	**0.038**	0.01	0.42
	Age	Adult	756	0.45	0.28	0.63	Chick	164	0.42	0.24	0.62	0.29	0.593	0.00	0.39
	Colony	Blomstrand	343	0.39	0.23	0.59	Krykkjefjellet	392	0.46	0.28	0.64	1.60	0.449	0.00	0.38
	Colony						Not recorded	185	0.47	0.28	0.66				
T. libellula	Stage	Incubation	147	0.04	0.01	0.09	Rearing	773	0.10	0.05	0.18	7.21	**0.007**	0.03	0.38
	Age	Adult	756	0.07	0.03	0.14	Chick	164	0.17	0.07	0.32	10.08	**0.001**	0.02	0.40
	Colony	Blomstrand	343	0.11	0.05	0.22	Krykkjefjellet	392	0.08	0.03	0.16	1.12	0.570	0.01	0.38
	Colony						Not recorded	185	0.08	0.03	0.18				
Atlantic species	Stage	Incubation	147	0.03	0.01	0.09	Rearing	773	0.10	0.03	0.22	16.76	**0.000**	0.03	0.55
	Age	Adult	756	0.09	0.03	0.20	Chick	164	0.05	0.01	0.14	3.61	0.057	0.01	0.50
	Colony	Blomstrand	343	0.09	0.03	0.21	Krykkjefjellet	392	0.09	0.03	0.20	0.34	0.845	0.00	0.49

	Colony						Not recorded	185	0.08	0.02	0.19				
Capelin	Stage	Incubation	147	0.01	0.00	0.03	Rearing	773	0.03	0.00	0.09	13.37	**0.000**	0.03	0.64
	Age	Adult	756	0.03	0.00	0.08	Chick	164	0.01	0.00	0.04	5.48	**0.019**	0.02	0.61
	Colony	Blomstrand	343	0.03	0.00	0.09	Krykkjefjellet	392	0.03	0.00	0.09	0.26	0.879	0.00	0.60
	Colony						Not recorded	185	0.02	0.00	0.08				
T. inermis	Stage	Incubation	147	0.21	0.11	0.35	Rearing	773	0.20	0.12	0.31	0.02	0.895	0.00	0.28
	Age	Adult	756	0.20	0.12	0.31	Chick	164	0.24	0.13	0.39	0.87	0.351	0.00	0.28
	Colony	Blomstrand	343	0.20	0.12	0.33	Krykkjefjellet	392	0.21	0.13	0.34	0.12	0.943	0.00	0.28
	Colony						Not recorded	185	0.20	0.11	0.33				
Total mass*	Stage	Incubation	142	8.0	6.3	10.1	Rearing	737	11.3	9.4	13.5	13.26	**0.000**	0.02	0.11
	Age	Adult	715	11.4	9.4	13.8	Chick	164	7.8	6.2	9.8	18.61	**0.000**	0.02	0.13
	Colony	Blomstrand	343	12.0	9.5	15.2	Krykkjefjellet	392	9.7	7.7	12.1	5.13	0.077	0.01	0.14
	Colony						Not recorded	144	11.4	8.6	15.2				

* Total mass is given as wet weight (g)

Table S4. Percentage point difference in most important diet categories if incubating birds were removed from the dataset. N_{inc} and N_{rear} columns indicate the number of incubating and rearing birds, respectively.

Year	N_{inc}	N_{rear}	Arctic species	Polar cod	*T. libellula*	Atlantic species	Capelin
1982	0	7	0.0	0.0	0.0	0.0	0.0
1983	0	7	0.0	0.0	0.0	0.0	0.0
1984	5	0	-40.0	-20.0	0.0	0.0	0.0
1987	0	22	0.0	0.0	0.0	0.0	0.0
1997	0	28	0.0	0.0	0.0	0.0	0.0
1998	0	11	0.0	0.0	0.0	0.0	0.0
2004	0	37	0.0	0.0	0.0	0.0	0.0
2005	7	129	0.4	0.3	0.1	-0.7	-0.7
2006	0	102	0.0	0.0	0.0	0.0	0.0
2007	12	45	-2.9	-2.9	0.0	4.7	4.7
2008	16	36	6.4	1.7	4.7	9.4	8.5
2009	35	29	-21.4	-21.8	-1.2	22.0	16.4
2010	17	71	-2.8	-3.8	2.1	1.0	1.0
2011	8	45	-0.4	-1.4	1.0	1.0	0.3
2012	9	66	2.2	0.4	1.6	1.2	0.5
2013	10	55	-0.1	-0.8	0.8	3.2	0.3
2014	8	16	4.2	-2.1	8.3	2.1	0.0
2015	18	24	1.8	0.0	1.8	-3.0	0.0
2016	2	43	1.0	0.9	0.1	1.7	0.6

Table S5. Percentage point difference in most important diet categories if chicks were removed from the dataset after removal of incubating birds. N_{adult} and N_{chick} columns indicate the number of adults and chicks, respectively.

Year	N_{adult}	N_{chick}	Arctic species	Polar cod	*T. libellula*	Atlantic species	Capelin
1982	7	0	0	0	0	0	0
1983	7	0	0	0	0	0	0
1984	0	0	0	0	0	0	0
1987	22	0	0	0	0	0	0
1997	28	0	0	0	0	0	0
1998	8	3	2.3	2.3	0	0	0
2004	31	6	-2.6	2.8	-4.9	0	0
2005	121	8	-0.5	-0.6	0.1	0.1	0.1
2006	60	42	-4.9	-5	-1.2	2.9	1.4
2007	43	2	0.5	0.5	0	-0.5	-0.5
2008	25	11	-2.3	5.4	-7.8	9.4	8.2
2009	27	2	-0.9	-1.1	0.3	3.8	3.1
2010	43	28	-8.2	0.8	-9.9	2.7	2.7
2011	33	12	6.7	7.3	-0.6	-0.6	-2.2
2012	44	22	-1.5	0	-2.3	3.8	7.6
2013	48	7	-1.2	-1.2	-0.5	1.2	0.3
2014	16	0	0	0	0	0	0
2015	24	0	0	0	0	0	0
2016	22	21	4	1.8	2.2	8.2	4.2

Table S6. Mixed effect model results on effects of explanatory variables on binary ("Bin") and wet weight ("Mass") diet data. "β" gives the slope of mixed effect models, "Min" and "Max" the 95% confidence intervals, "Np" the number of model parameters, "Df" the residual degrees of freedom, "χ^2" the Chi-square test value, "P" the corresponding *p* value, R^2_m marginal R^2 value and R^2_c conditional R^2 value.

Diet item	Variable	Data	β	Min	Max	Np	Df	χ^2	P	R^2_m	R^2_c
Arctic species	Sea ice index	Bin	0.22	0.13	0.32	8	912	15.7	**0.000**	0.15	0.36
Arctic species	Sea ice index	Mass	0.17	0.11	0.22	6	709	20.0	**0.000**	0.18	0.31
Arctic species	Temperature	Bin	-0.59	-1.33	0.14	8	890	2.4	0.124	0.05	0.40
Arctic species	Temperature	Mass	-0.36	-0.77	0.06	6	709	2.8	0.092	0.04	0.32
Arctic species	Population size	Bin	-0.03	-0.07	0.01	8	807	1.8	0.178	0.09	0.43
Arctic species	Population size	Mass	-0.02	-0.03	0.00	6	647	2.5	0.113	0.05	0.37
Polar cod	Sea ice index	Bin	0.20	0.11	0.29	8	912	14.2	**0.000**	0.13	0.34
Polar cod	Sea ice index	Mass	0.18	0.13	0.22	6	873	19.2	**0.000**	0.20	0.31
Polar cod	Temperature	Bin	-0.72	-1.44	-0.01	8	890	3.8	0.052	0.07	0.42
Polar cod	Temperature	Mass	-0.35	-0.75	0.05	6	873	3.0	0.081	0.05	0.32
Polar cod	Population size	Bin	-0.03	-0.07	0.01	8	807	2.1	0.145	0.08	0.42
Polar cod	Population size	Mass	-0.02	-0.03	0.00	6	809	2.8	0.095	0.06	0.35
Atlantic species	Sea ice index	Bin	-0.34	-0.56	-0.12	8	912	12.0	**0.001**	0.24	0.56
Atlantic species	Sea ice index	Mass	-0.05	-0.12	0.02	6	709	2.0	0.157	0.03	0.33
Atlantic species	Temperature	Bin	0.93	0.12	1.75	8	890	5.1	**0.024**	0.10	0.51
Atlantic species	Temperature	Mass	0.23	-0.07	0.53	6	709	2.2	0.142	0.03	0.33
Atlantic species	Population size	Bin	0.02	-0.02	0.05	8	807	1.0	0.321	0.03	0.50
Atlantic species	Population size	Mass	0.00	-0.01	0.02	6	647	0.2	0.632	0.01	0.36
Total mass	Sea ice index	Mass	0.05	0.02	0.08	6	709	9.7	**0.002**	0.05	0.14
Total mass	Temperature	Mass	-0.09	-0.26	0.08	6	709	1.2	0.268	0.01	0.15
Total mass	Population size	Mass	0.00	-0.01	0.01	6	647	0.3	0.614	0.00	0.15

References

1. Lidster, W. W., Lilly, G. R. & Dawe, E. G. Otoliths of Arctic cod (*Boreogadus saida*), small Atlantic cod (*Gadus morhua*), and three other fish species from Newfoundland waters: description and relationship of body length to otolith length. *J. Northwest Atl. Fish. Sci.* **16,** 33–40 (1994).

2. Falk-Petersen, I.-B., Frivoll, V., Gulliksen, B. & Haug, T. Occurrence and size/age relations of polar cod, *Boreogadus saida* (Lepechin), in Spitsbergen coastal waters. *Sarsia* **71,** 235–245 (1986).

3. Lønne, O. J. & Gulliksen, B. Size, age and diet of polar cod, *Boreogadus saida* (Lepechin 1773), in ice covered waters. *Polar Biol.* **9,** 187–191 (1989).

4. Gjøsæter, H. & Ajiad, A. M. Growth of polar cod, *Boreogadus saida* (Lepechin), in the Barents Sea. *ICES J. Mar. Sci.* **51,** 115–120 (1994).

5. Nahrgang, J. *et al.* Gender specific reproductive strategies of an arctic key species (*Boreogadus saida*) and implications of climate change. *PLoS One* **9,** e98452 (2014).

6. Prozorkevich, D., Skaret, G. & Alvarez, J. Pelagic fish abundance and distribution. In *Survey report from the joint Norwegian/Russian ecosystem survey in the Barents Sea and adjacent waters, August-October 2016* (eds. Prozorkevich, D. & Sunnanå, K.) 1–21 (IMR/PINRO Joint Report Series, No. 1, 2017).

7. Winters, G. H. Life history and geographical patterns of growth in capelin, *Mallotus villosus*, of the Labrador and Newfoundland areas. *J. Northwest Atl. Fish. Sci.* **3,** 105–114 (1982).

8. Loeng, H. & Gjøsæter, H. Growth of 0-group fish in relation to temperature conditions

in the Barents Sea during the period 1965-1989. *ICES J. Mar. Sci.* **49,** 1–9 (1990).

9. Eriksen, E., Ingvaldsen, R., Stiansen, J. E. & Johansen, G. O. Thermal habitat for 0-group fish in the Barents Sea; how climate variability impacts their density, length, and geographic distribution. *ICES J. Mar. Sci.* **69,** 870–879 (2012).

10. Ottersen, G. & Loeng, H. Covariability in early growth and year-class strength of Barents Sea cod, haddock, and herring: the environmental link. *ICES J. Mar. Sci.* **57,** 339–348 (2000).

11. Michalsen, K., Ottersen, G. & Nakken, O. Growth of North-east Arctic cod (*Gadus morhua* L.) in relation to ambient temperature. *ICES J. Mar. Sci.* **55,** 863–877 (1998).

12. Breiby, A. *Otolitter fra saltvannsfisker i Nord-Norge*. (Universitetet i Tromsø, Institutt for Museumsvirksomhet, 1985).

13. Gjösæter, J. Age, growth, and mortality of the myctophid fish, *Benthosema glaciale* (Reinhardt), from Western Norway. *Sarsia* **52,** 1–14 (1973).

14. Killick, R. & Eckley, I. changepoint: An R Package for changepoint analysis. *J. Stat. Softw.* **58,** 1–15 (2014).

15. Hinkley, D. V. Inference about the change-point in a sequence of random variables. *Biometrika* **57,** 1–17 (1970).

16. Bates, D., Mächler, M., Bolker, B. & Walker, S. Fitting linear mixed-effects models using lme4. *J. Stat. Softw.* **67,** 1–48 (2015).

17. R Core Team. R: A language and environment for statistical computing. R Foundation for Statistical Computing, Vienna, Austria (2017).

18. Bolker, B. M. *et al.* Generalized linear mixed models: a practical guide for ecology and evolution. *Trends Ecol. Evol.* **24,** 127–135 (2009).

19. Barton, K. MuMIn: Multi-model inference. R package version 1.15.6 (2016).

20. Johnson, P. C. D. Extension of Nakagawa & Schielzeth's R^2_{GLMM} to random slopes models. *Methods Ecol. Evol.* **5,** 944–946 (2014).

21. Bolker, B. *Ecological models and data in R*. (2008).
